# Temporary Anchorage Devices

**DOI:** 10.7759/cureus.44514

**Published:** 2023-09-01

**Authors:** Shalabh Baxi, Virag Bhatia, Anand Tripathi, Mangleshwar Prasad Dubey, Pratiksha Kumar, Sagar Mapare

**Affiliations:** 1 Department of Orthodontics, Government Dental College, Raipur, Raipur, IND; 2 Department of Orthodontics, Government College of Dentistry, Indore, IND; 3 Department of Orthodontics, Saraswati-Dhanwantari Dental College and Hospital, Parbhani, IND; 4 Department of Orthodontics, Guru Gobind Singh College of Dental Sciences and Research Centre, Burhanpur, IND; 5 Department of Oral Pathology and Microbiology, Government College of Dentistry, Indore, IND; 6 Department of Orthodontics, Dr. Hedgewar Smruti Rugna Seva Mandal's Dental College and Hospital, Hingoli, IND

**Keywords:** orthodontics, orthodontic bone screws, anchorage, mini screw, temporary anchorage devices

## Abstract

Reaction to the force application is observed in the clinical scenarios as anchorage loss, which is the unwanted movement of the teeth. A plethora of approaches have been developed over time in orthodontics to overcome anchorage loss. These approaches are termed anchorage reinforcement procedures. Anchorage loss refers to the unintended movement or shifting of teeth that are intended to remain stable and serve as anchoring points during orthodontic treatment. This loss of stability can occur in various dimensions, including horizontal, vertical, or transverse, and can result in undesired changes to the overall positioning and alignment of teeth. Anchorage can be termed as conventional intraoral anchorage which usually leads to significant anchorage loss. The conventional extraoral anchorage such as headgear suffers from the issue of compliance.

## Introduction and background

In orthodontics, a temporary anchorage device (TAD) is one that is placed on the bone for a limited amount of time in order to improve anchorage in one of two ways: (i) by providing support for the teeth of the reactive unit (indirect anchorage) or (ii) by eliminating the need for the reactive unit (direct anchorage), and is then removed. Mini-implants also called mini-screws or TADs in orthodontics. Orthodontic mini-implants are smaller in dimensions compared to prosthetic dental implants. The size of orthodontic mini-implants varies from 1.5mm to 2mm in diameter and from 6mm to 10mm in length. The surfaces of orthodontic mini-implants are polished and smoother compared to prosthetic dental implants. The reason is that orthodontic mini-implants do not depend on osseointegration with bone but rather on mechanical retention for retention [1].

Historical perspective

The evolution of orthodontic implants followed the development of dental implants and orthognathic fixation methods. These techniques merged biological and biomechanical principles with osseointegration. Gainsforth and Higley (1945) first proposed the idea of skeletal anchorage using Vitallium screws in a dog's ramus for distalizing a maxillary canine. The concept of osseointegration and titanium implants for tooth replacement was introduced by Per Ingvar Branemark. Creekmore and Eklund (1983) reported the first clinical use of TADs for anterior nasal spine intrusion. Recent research explores palatal on plants, mid-palatal screws, and miniplate implants for orthodontic applications [2].

More recently, a good middle ground was reached with the introduction of orthodontic bone screws (OBS), which not only had an extra-radicular site of placement in the infra-zygomatic crest (IZC) of the maxilla and the buccal shelf (BS) area of the mandible but also did not require extensive surgical intervention for their placement, resulting in significantly lower failure rates than regular mini-implants [2].

Components of TADs

Head

The head bears the springs and elastics are attached to the screw at the end that is accessible to the mouth. For use with a mini-screw driver, it features a groove or other shape that accommodates a screwdriver. For various anchoring methods and to avoid irritating soft tissue, a wide variety of head designs are offered. The most popular form has the form of a button and might be a sphere, a double sphere, or a hexagon. For direct anchoring, a screw with a hole of 0.8 mm in diameter in the head or neck is typically utilized.

Neck

The neck acts as the transmucosal section of the screw and links the screw to the head. The neck length may be adjusted to accommodate a range of mucosal thicknesses. Plaque collection around the neck may be reduced by maintaining a smooth, well-polished surface on the neck. Most implant failure due to peri-implantitis often originates at the intersection of TAD with the mucosa [3-6].

Screw Part

The screw part is implanted, and it becomes a permanent component of the cortical or medullary bone. The TAD's threaded screw around the shank or main body has a cutting edge that makes it easier to install [7]. The torque needed for insertion is proportional to the tension created during insertion, and stress levels are in turn determined by the depth and angle of the cutting edge. Orthodontic mini-implants have a parallel thread that tapers only at the end, whereas mini-screws have a conical thread. TAD length is defined as the overall threaded body length. Based on the patient's anatomy, it might be anywhere from 5mm to 12mm [8].

Orthodontic practitioners may greatly benefit from using mini-implants, also known as mini-screws, as an absolute anchoring method. New locations, called extra-alveolars, have been proposed despite the fact that they are often placed in the alveolar process between the roots of adjacent teeth. Many orthodontic treatments call for an effective and secure anchoring system, and many writers have suggested the IZC and the mandibular BS as good locations.

## Review

Size and dimensional variations of TADs: Micro-implants and bone screws

While both micro-implants and extra-radicular bone screws (IZC, BS) fall under the category of temporary anchorage devices, the former are placed closer to the roots of the teeth (intra-radicular) and the latter are placed further away from the roots (extra-radicular) in the infra-zygomatic regions of the maxilla and the BS regions of the mandible. However, they serve the same function in the body by providing a skeletal anchor [3]. Bone screws, on the other hand, may be anywhere from 10 to 14 millimeters in length and have a minimum diameter of 2 millimeters, whereas the standard size range for a micro-implant is 6 to 11 millimeters in length and 1.3-2 millimeters in diameter, again depending on the clinical circumstance it has to be utilized for. Bone screws, like micro-implants, may have either a short or long head, with the choice being made based on the anatomical location and the specific clinical scenario. Even the form of their caps may vary, with the mushroom shape being the most prevalent.

Classifications

TADs are categorized based on various size parameters, head types, screw types, and biocompatibility. These factors influence their selection and placement in orthodontic treatment. The Cope JB classification (2005) further categorizes TADs into biologic, ankylosed teeth, dilacerated, and biocompatible types [3].

Implant Size Classifications

TADs are classified by size, considering their length and diameter. The sizes are typically categorized as small, medium, and large based on the following parameters:

Length: TADs range from 4 to 12 mm in length. Their classification as small, medium, or large corresponds to this range, indicating the extent of penetration into bone.

Diameter: TADs exhibit diameters of 1.15 to 2.5 mm. Small, medium, or large categories are assigned based on the diameter, indicating the overall thickness of the implant.

Head Type Classifications

TADs possess different head types, influencing their function and integration into orthodontic mechanics. Head types are characterized as follows:

Small head: TADs with small heads are discreet and suitable for limited space scenarios.

Long head: TADs with long heads offer increased surface area for force distribution, enhancing stability.

Circle head: TADs with circle heads provide versatility for force application from various angles.

Fixation head: Fixation heads aid in securing auxiliary components, and supporting complex orthodontic movements.

Bracket head: Bracket heads allow direct attachment of archwires, mimicking traditional brackets for force application.

Hook head: Hook heads provide attachment points for elastics or other auxiliary elements, expanding treatment possibilities.

Screw Type Classifications

TADs are classified based on their screw type, which determines their insertion mechanism and efficacy:

Self-drilling: Self-drilling TADs incorporate a drill bit and can penetrate bone without pre-drilling.

Self-tapping: Self-tapping TADs create threads as they are inserted, aiding in stable fixation.

Thread-forming: Thread-forming TADs shape bone as they are inserted, optimizing implant stability.

Thread-cutting: Thread-cutting TADs engage with bone threads to enhance anchorage.

Selection of Implant Locations

TADs can be placed in different locations within the oral cavity to achieve specific treatment goals. Some common implant locations include:

Midpalatal implants: Midpalatal implants were desirable due to the strong primary stability gained through the exploitation of cortical bone, whereas buccal implants were less appealing due to their less dependable stability in the past. The disadvantages include that midpalatal implants might be visible in certain cases, causing aesthetic concerns for some patients. The surgical procedure for midpalatal implant placement can be more intricate compared to some other implant locations. Depending on the implant size and location, there might be potential interference with the tongue's movement and function.

Palatal interdental implants: Positioned in the interdental space on the palate, these implants provide control over force application. The disadvantages of palatal interdental implants include the potential for neighboring tooth mobility to be inhibited and the need for a transpalatal arch (TPA).

Buccal interdental implants: Placed in the buccal interdental space, these implants are relatively simple to integrate into treatment. However, the buccal interdental space might be restricted in some cases, making implant placement challenging. In the past, buccal implants were considered to have less dependable stability due to potential challenges with bone quality and quantity.

Indications

Indications include absolute anchorage in case of high angle backward maxillary plane (BMP), failed headgears, cases of missing teeth, first molars, difficult tooth movements, anterior/posterior intrusion, en masse distalization of U/L arches, molar uprighting, molar distalization, adult orthodontics, orthopedic traction.

Contraindications

In cases of systemic bone illnesses and other medically compromised states, orthodontic implants are not recommended. Patients under the age of 12 who have not finished their skeletal development should not have a TAD inserted. Mini-screws shouldn't be used in regions where the bone is reshaping itself, such as a mending socket or around a baby tooth. Mini-screws cannot be placed in bone with a cortical thickness of less than 0.5mm. An experienced clinician is essential for successful implant placement. The intrusive nature of the operation raises ethical concerns.

TAD placement protocol

The patient's medical history and evaluation of the patient's appropriateness for the extra surgery of mini-screw installation are crucial. Gingival inflammation and periodontal disease must be absent from the patient's mouth before treatment can begin. He or she should be self-motivated and skilled enough to take the additional steps necessary to keep the mouth clean, particularly around the mini-screw. Intraoral radiographs of the prospective mini-screw site(s) are taken in addition to standard orthodontic records to evaluate factors such as interradicular bone breadth, crestal bone loss, root length, and mesiodistal angulation. The treatment to be performed, the expected course of events, and any potential problems related to this style of therapy should all be thoroughly described to the patient and his or her parents. Consent with full understanding should always be sought. A bone density test is necessary if there are concerns about bone quality on regular X-rays, a medical history, or a history of medication that might alter bone metabolism. CT scans and cone beam computed tomography (CBCT) may provide site-specific estimates of bone density. Implants may not work well with bone types D4 and D5, as has been speculated [4].

The anatomical constraints of its location and the desired biomechanics dictate the size of the mini-screw implant (MSI) that may be used. Mini-screw length is determined by buccolingual/buccopalatal dimensions of the alveolus or nasopalatal thickness of the palate, whereas mini-screw diameter is determined by the amount of interradicular bone available. Implants of standard length are utilized in the interradicular bone of the buccal surface of the maxilla and mandible, whereas longer implants are used in the retromolar region. The front palate is better suited to implants with a bigger diameter but shorter length. Mini-screw dimensions for different orthodontic applications are shown in Table 1.

**Table 1:** Recommended mini-screw dimensions for different orthodontic applications MSI: mini-screw implant

Purpose/Region	Recommended Mini-Screw Dimensions	Explanation
Front Teeth Retraction	Diameter: 1.4-1.5 mm Length: 7-8 mm	Effective for mass retraction between second and first maxillary premolars.
Intraoral Molar Distalization	Diameter: 1.4-1.6 mm Length: 8 mm	Optimal for molar distalization between first and second premolar roots.
Insertion between Roots	Diameter: ≥ 1 mm of bone around max diameter	Suitable for placement between roots, ensuring sufficient bone support.
Trabecular Bone (e.g., retro-molar region)	Diameter: 2 mm Length: 10 mm	Longer screw for insertion into trabecular bone.
General Force Range	≤ 300 g	Most orthodontic applications require forces within this range.
High-Resistance Cases	Diameter: 1.2-1.3 mm Force: Up to 500 g	1.2-1.3 mm diameter screws may resist forces up to 500 g.
Wider Diameter for More Force	Diameter: 1.4-1.6 mm	Use a wider screw if more force is needed and there's sufficient room between the roots.
Neck Height Matching Mucosal Thickness	Utilize MSI with matching neck height	If possible, choose an MSI with neck height corresponding to mucosal thickness.

MSI installation is a brief and seemingly minimal invasive surgery, but it requires extreme accuracy and care to be carried out successfully. A self-drilling screw's placement and angle are defined by a thorough clinical evaluation and analysis of a recent intraoral periapical X-ray. The night before surgery, the patient should take 250 milligrams of amoxicillin or another appropriate antibiotic, and those who seem to be less tolerant of pain should be given a safe painkiller an hour beforehand. Plaque and tartar are removed from the mouth carefully, with specific attention paid to the area where mini-screws will be inserted. The prescribed treatment consists of a 1-minute mouth rinsing with 10 milliliters of 0.12% chlorhexidine gluconate mouthwash. The surgery can be performed with a 15% topical anesthetic, but we've found that infiltrating the area with 0.5 mL of local anesthesia gives us the most peace of mind. Before beginning MSI implantation, the patient is made comfortable and their fears are allayed. After the MSI site has been marked, preselected MSI is used to begin the insertion procedure. A dent is formed in the cortical bone using a 0.9 mm round bur at 300-500 rpm and generous saline irrigation for screws that do not need drilling. Mini-screw performance may be negatively impacted by the heat produced while drilling, however, irrigation can mitigate this issue. Then, using the proper driver, the mini-screw is gently inserted into the desired location at the specified angle [5]. A buccal screw is positioned between the roots of the premolars and molars at an angle of 45° to 60° with respect to the long axis of the teeth in the maxilla. In the back of the mandible, the insertion angle is reduced to 10-30 degrees. After the surgery is finished, an intraoral periapical (IOPA) radiograph is done to verify the position of the mini-screw. In the first few days after surgery, you should take your antibiotics as prescribed and practice rigorous oral hygiene to protect your mini-screw. When necessary, use the pain relievers. After a week has passed, a clinical evaluation of the implant's mobility and a thorough examination of any unusual symptoms of inflammation should be performed. Inflammation and a limited range of motion at the neck should be expected after a failed mini-screw. The patient should use a gentle brush to clean the area around MSI after each meal. You should gargle with chlorhexidine mouthwash (0.12%, 10 mL) at least twice a day. The patient has to know what to do if his or her mobile MSI causes pain or discomfort: contact their orthodontist right away.

The mechanical locking in the bone by MSIs is thought to offer anchoring even though they are not anticipated to osseointegrate. As a result, the majority of medical professionals advise starting mild loads on the MSIs right away. However, Dr. Kharbanda's suggestion of waiting two to three weeks before loading an MSI is the standard procedure. When the MSI (self-tapping or self-drilling) is drilled, it causes damage to the bone, which includes compression, strains, and microfractures, as well as the accumulation of bone debris and the release of inflammatory mediators. The AIIMS Department of Orthodontics has conducted research on peri-mini-screw crevicular implant fluid (PMICF) to evaluate implant stability and peri-implant inflammation. Inflammatory markers were detected in PMICF after 1 and 24 hours after MSI insertion and loading, according to the studies. Inflammatory indicators return to baseline throughout a three-week time span. Thus, Dr. Kharbanda's procedure involves a three-week wait before the first loading phase begins [6-8].

Mini-screws may be removed under topical anesthesia by first gripping the head with tweezers or another suitable device, then using the instrument used for driving, that is MSI driver, to make a firm but gradual anticlockwise motion. Mini-screw wounds do not need systemic antibiotics or pain relievers, nor do they need sutures or post-operative dressings. In 3-5 days, the wound will have healed on its own, with mucosa covering the area.

Biomechanics and tooth movement with TADs

Anterior torque control, canine axis control, and vertical control of the anterior teeth are all important biomechanical factors in the successful retraction of the front teeth to their appropriate position. Anteroposterior anchoring management is no longer a challenge because of the availability of orthodontic mini-implants. Anterior brackets with increased tension applied to the labial crown may be prescribed according to the desired degree of retraction [9]. Prior to anterior retraction, a cephalometric radiograph should be taken to assess the quantity of accessible alveolar bone and root morphologies. Even with significant amounts of anterior retraction, root resorption is less likely if there is enough alveolar bone towards the apex. Root resorption is more likely to occur with the same amount of anterior retraction if there is inadequate alveolar bone. It is interesting to note that by adjusting the position of the temporary skeletal anchorage device (TSAD) in relation to the occlusal plane and by altering the hook length from where the force originates, we can manipulate individual segments of the arch as well as the final tooth movement [10,11]. Low-pull, medium-pull, and high-pull mechanics are used to place the implant in the bone at different depths relative to the archwire. Implants positioned 8-10 mm above the archwire (12-14 mm above the occlusal plane) are considered to have medium-pull mechanics. Anything higher than this would be a high-pull mechanic and lower than 8 mm would be termed as low-pull mechanics.

High-Pull Mechanics

The high pull mechanism is often employed to achieve vertical control of the dentition or the craniofacial structures. This mechanism involves the application of forces in the vertical direction to either intrude or extrude specific teeth or groups of teeth, depending on the treatment goal. When vertical forces are directed toward the bone, they can lead to the intrusion (downward movement) of specific teeth. This is useful in cases where overeruption of teeth has occurred or when certain teeth need to be aligned with the occlusal plane. Conversely, vertical forces directed away from the bone can cause extrusion (upward movement) of teeth. This is employed when certain teeth need to be brought into alignment with adjacent teeth or to counteract deep bites. The high pull mechanism with TSADs can be highly effective in achieving specific treatment goals, it requires careful treatment planning, skillful execution, and thorough patient monitoring. Additionally, patient compliance in terms of maintaining proper oral hygiene and following the orthodontist's instructions is crucial to the success of the treatment.

Low-Pull Mechanics

The upper occlusal plane as a whole would be steepened by this force vector, and the moment it produces will be clockwise. This is advantageous in a dental open bite scenario, but a posterior open bite develops in such cases. If low-pull mechanics are used in both arches, posterior open bite develops due to rotation of occlusal planes. If anterior retraction along with some intrusion is desired, then either of the strategies could be employed. To obtain a force vector along the center of resistance (C. Res.), a high-pull implant could be used at the distal to the second premolar. For more intrusion, the implant location could be moved anteriorly into the extraction space.

Different types of tooth movements

Controlled Tipping

Short hook placement and line of force should be below the crest. Additional labial crown torque for intrusion and root movement by mini-screw placement in the anterior region for additional labial crown torque.

Translation

When low-pull mechanics are used posterior open bite needs to be negated by using box elastics in the posterior region. Some authors have also recommended the use of anterior bite planes to resist bite-deepening tendencies in anterior teeth. In high pull mechanics, if the line of force passes above the C. Res. of dentition there would be a mesial drag on molars without even attaching any retraction force to the molar hook. This can be prevented by placing a TPA. In the same situation, an anterior open bite tendency would exist and needs to be countered using anterior box elastics. Another important biomechanical consideration is the symmetric location of the TSADs during anterior retraction. Force vectors change if the screw location is asymmetric and can cause canting of the occlusal plane. This has to be avoided as far as possible. However, in some situations where the failure of the TSAD occurs and the new screw is placed in a different position (vertically), necessary care in the form of a single cantilever intrusion spring on the side of the lower implant becomes necessary to prevent canting [9].

Molar Distalization

The posterior boundaries of distalization are defined by the tuberosity, the most posterior cortical bone in the maxilla, and the most posterior lingual cortical bone in the mandible. Prior to distalization, it is important to assess whether or not the molar can be moved excessively. Determine whether or not third molar extraction is necessary before proceeding with other treatments, taking into account the amount of free space and the risk of third molar impaction. In order to cause motion in the body, distal force must be supplied in a three-dimensional fashion via the molars' point of resistance. But from a clinical standpoint, it just can't happen. Control of the second molar is crucial because, when seen in three dimensions, force may be given away from the point of resistance, causing the teeth to rotate in three dimensions. The other teeth are affected because the root surfaces of the first and second molars are so large and hard to manage. It is recommended to distalize the second molars initially, either individually or in groups. In other words, the second molars should be the primary targets of distalization pressures. The ability to manipulate the second molar in all three dimensions is fundamental from a biomechanical standpoint [10].

Sequential Orthodontic Mechanics

Molar distalization is achieved by using an open coil spring to the molar and the other end is activated by, connecting a ligature to the TSAD. This distal movement would necessarily include some bodily movement and some distal tipping. After molar distalization is achieved, separate retraction of the anterior as well as premolar segment is initially started taking support from the same TSAD using a closed coil spring. During this stage, distal vertical elastics are used to control the angulation of the molar which would have tipped distally. The TSAD may, however, have to be changed after a while since during retraction, the roots of the second premolar come very close to the screw.

Molar Protraction

Similar to molar distalization, molar rotation may take place in three dimensions if the force acting on the teeth is directed away from the tooth's C. Res. The protractive force on a labial hook causes a tendency toward tipping mesially. Apical placement of mini-implants may lead to intrusion of the posterior teeth because the protractive force has an intrusive force vector. Occlusal canting occurs when there is a unilateral intrusion. There must be vertical and tipping control. An opposing curve may counteract the mesial lean. However, vertical control cannot be achieved using this approach. The negative consequences of mesial tilting and incursion may be lessened by using a strong wire heavier than 0.017 x 0.025-inch SS. When protraction is extended and fine control is unavailable, this is not a foolproof method of keeping out intruders. Both issues may be easily fixed by inserting a lever arm into the first molar's auxiliary tube [12-14].

In cases where posteriors are to be protracted (Group C arches), implants can be placed between canine and premolar. The TSAD's coil spring is first used to provide force for protracting the first molar. There will come a time when the molar tips forward in conjunction with its mesial migration. Once the majority of the first molar has been protracted, a tip-back bend is applied to elevate it (along with a ligature consolidation from premolar to molar), and the coil spring is then joined to the second molar. While the second molar is being prolonged, the tip-back aids in righting the first molar's root. After the first molar completely uprights, its band is removed, and the same wire will serve to upright the second molar too. Since the protraction force to the molars is being applied from the buccal side, there will be a corresponding mesial-in rotation tendency. The archform in the wire should therefore be suitably contoured inwards from the premolar region [15,16].

In adult orthodontic cases, along with supra-eruption, the other frequently occurring problem is mesially angulated molars, which act as unfavorable abutments for a fixed bridge, even if implant prosthesis is to be considered, the space available becomes inadequate and hence, uprighting becomes necessary.

Clinical applications

Anterior Intrusion

It's easy and efficient to utilize implants to provide a single force directly, but you should never use an excessive amount of force to break in. The intrusive force from an implant is typically constant and unchanging, but force systems between the bracket and wire do fluctuate, therefore adding a single-force component from implants may boost treatment efficiency. Unwanted results may occur after introducing continuous arch mechanics [17].

Positions of Implants for Direct Intrusive Force Application

When one implant is positioned between the central incisors, it is possible to apply an invasive force. However, labial flaring is thought to result from the force system created by these mechanisms. Intruding the six front teeth and regulating the canine axis are both improved by implants placed on the mesial side of the canines. Implants placed on the distal aspect of the canines are beneficial because they increase the retractive force vector.

Anterior Extrusion

Both anterior intrusion and extrusion need three-dimensional management of the teeth using the same biomechanical principles. In addition to the vertical position, the axis and anteroposterior location of the incisal edges should be managed. Torque regulation is also crucial. The anteroposterior positioning of the root apices may be exacerbated by the conventional practice of continual full-arch leveling, which can lead to extrusion with uncontrolled tipping.

Posterior Intrusion

Intruding posterior teeth is one of the biggest advantages that TSADs offer. Due to the complexity of the molars' several big roots and the need for a strong alveolar bone response, posterior intrusion is one of the most challenging tooth motions to complete in developing an occlusal treatment strategy. Occlusal plane shifts may be loosely divided into two categories from a clinical perspective. (a) The term “parallel intrusion” describes a situation when both the molars and premolars have been pushed forward. A “gummy” grin or a lengthy face can only be fixed by doing this. (b) When the second molar regions are more heavily intruded than the premolar regions, this is known as a nonparallel intrusion. Unwanted consequences might lead the opposite to occur. The more challenging goal of correcting an open bite requires nonparallel penetration. The recommended measure is to use screws on both buccal and palatal aspects as depicted. Since the moments on either side, cancel out each other, a pure intrusion is obtained [18-21].

Molar Intrusion

Check the alveolar bone to rule out infrabony pockets. Crest of molar and center of the occlusal table close to the palatal root. Two mini-screws should be placed at the buccal-mesial interdental area, and palatal-distal interdental area, severe extrusion uses three or four mini-screws [16].

Posterior Extrusion

The implant mechanics make extrusion more challenging than intrusion, which is the opposite of how normal mechanics work. Extrusion, like incursion, requires three-dimensional control of the molar. Physical extrusion requires the application of a force through the C. Res. Lingual tipping is caused only by buccal extrusive force; hence you need to apply force from both the buccal and lingual sides to achieve torque control. When it comes to extrusive mechanics, implants tend to fall short. Extrusive mechanics call for the employment of push springs that extend from implants. A point of contact between the moving components is required if push springs are to be employed.

Occlusal Cant Correction

Dental occlusal cants can be corrected with TSADs readily; this feature has a significant advantage over all other intra-oral mechanisms used earlier. Depending on the situation, either a single TSAD could be used or two - one in the upper and the other on the opposite side in the lower arch. This correction should be done on a sufficiently thick rectangular wire (19X25 SS in 0.022” slot) to prevent individual tooth movements along the arch [14].

Maxillary Orthopedic Expansion

Unwanted tooth movement (buccal tipping) during expansion is the first traditional issue with maxillary expansion, followed by the question of how to accomplish midpalatal suture separation in adult patients, and finally, the issue of stability [22]. The skeleton anchorage might be advantageous in situations like these. However, the technique for maxillary orthopedic expansion using mini-implants needs additional study before it can be used.

Asymmetric Transverse Control

The scissor bite is a two-dimensional issue, appearing both horizontally and vertically. To correct the asymmetry of a molar's location, a passive TPA with extension arms may also be used.

Challenges and failures

Immediate failure as loosening during the initial healing phase, improper insertion sites, rare cortical bone, recent extraction sockets, redundant overlying soft tissue, improper handling-wobbling, or abrupt change path of insertion. Delayed failure is caused by excessive loading, sudden impact during mastication, root contact, and excessive or insufficient bone remodeling [23].

Root damage if occurs then removal of the mini-screw should be done. Periodontal ligament (PdL) contact may not cause discomfort or pain.

Mini-screw fractures are more likely to occur when the diameter of the mini-screw is less than 1.5 mm. These fractures can result from several factors, including the removal of bone around the mini-screw thread during placement. It's important to note that any resulting discomfort or pain is primarily attributed to irritation of nerve endings within the soft tissue and periosteum, rather than originating from the actual bone itself. In cases where mini-screw fractures occur and discomfort ensues, a suitable approach is to recommend the use of non-steroidal anti-inflammatory drugs (NSAIDs) for a period of two days.

Future perspectives of TADs

Miniaturization and Design Improvements

TADs may become smaller and more ergonomic, minimizing patient discomfort during placement and reducing soft tissue irritation [2,11].

Smart TADs

Integration of technology could lead to "smart" TADs equipped with sensors, enabling real-time monitoring of force application and tooth movement.

Material Innovations

Research into novel materials could lead to TADs with enhanced biocompatibility, strength, and durability.

Customization and 3D Printing

Personalized TADs designed through 3D printing could optimize treatment outcomes by tailoring devices to individual patient anatomy.

Improved Techniques

Future research might unveil refined techniques for optimal TAD placement and biomechanics, maximizing treatment success.

Multidisciplinary Integration

TADs could find applications beyond orthodontics, collaborating with other dental specialties for comprehensive treatment approaches.

Patient-Centric Focus

Future TADs could prioritize patient comfort and convenience, with smoother insertion procedures and reduced treatment times.

The contemporary applications of TADs have significantly expanded the scope of orthodontic treatment, offering solutions to complex cases. Looking ahead, the future of TADs holds exciting prospects for enhanced device design, advanced techniques, and interdisciplinary collaboration, ultimately shaping the landscape of orthodontics and patient care.

## Conclusions

TADs, also known as mini-screws or mini-implants, are small devices used in orthodontics to provide additional anchorage and support during tooth movement. They are placed on the bone for a limited period and then removed. TADs are smaller than traditional dental implants, ranging from 1.5mm to 2mm in diameter and 6mm to 10mm in length. Unlike traditional dental implants, TADs do not rely on osseointegration but instead use mechanical retention to provide support.

TADs also have been included in the envelope of discrepancy to avoid orthognathic surgery in borderline cases and increase the range of treatment possible in conjunction with conventional fixed appliances. They are particularly useful for achieving absolute anchorage, addressing anchorage loss issues, and enabling specific tooth movements such as anterior retraction, posterior distalization, molar protraction, molar uprighting, and midline correction.
